# Impact of an exercise and health education program for health promotion in textile workers: a pre-post intervention study protocol

**DOI:** 10.3389/fpubh.2026.1856664

**Published:** 2026-06-02

**Authors:** Carolina Jiménez-Sánchez, Paula Cordova-Alegre, Daniel Sanjuan-Sánchez, Garazi Ibiricu-Abendaño, Juan Rabal-Pelay, Eduardo Piedrafita

**Affiliations:** 1 Faculty of Health Sciences, Universidad San Jorge, Zaragoza, Spain; 2 Faculty of Nursing and Physiotherapy. University of Lleida, Lleida, Spain

**Keywords:** digital health, exercise therapy, health education, occupational health, textile workers, workplace intervention, work-related musculoskeletal disorders

## Abstract

Work-related musculoskeletal disorders are highly prevalent among manufacturing workers and represent a major cause of pain, functional limitation, reduced work ability and productivity loss, particularly in textile settings characterized by repetitive upper-limb tasks and sustained postures. Despite recommendations supporting exercise and education as first-line strategies, scalable workplace-embedded programs remain insufficiently implemented. This study aims to evaluate changes associated with an eight-week multimodal intervention integrating health education and app-supported exercise in textile workers. A quasi-experimental single-arm pre–post study will be conducted in a textile manufacturing company in Zaragoza, Spain, including workers aged 18–65 years. The intervention will consist of weekly face-to-face educational workshops and a twice-weekly digitally supported exercise program. Outcomes will be assessed at baseline, post-intervention and one-month follow-up. The primary outcome will be change in musculoskeletal pain intensity, and secondary outcomes will include musculoskeletal symptoms, physical function, physical activity, work ability, sleep quality, psychological distress, health-related quality of life and work productivity. Data will be analyzed using paired statistical tests according to distribution, with a significance level of 0.05. The study has received ethical approval from the Universidad San Jorge Ethics Committee and the Research Ethics Committee of Aragón, and findings will be disseminated through peer-reviewed publications and scientific conferences.

**Clinical trial registration:**

ClinicalTrials.gov, identifier (NCT07360626).

## 1 Introduction

Musculoskeletal disorders (MSD) are among the most prevalent causes of disability worldwide and exert a profound impact on individual well-being, healthcare systems and labor productivity across diverse occupational sectors (1–3)These conditions frequently lead to chronic pain, functional limitations and reduced quality of life, contributing substantially to work absence, presenteeism and long-term disability (2, 4) Within occupational environments, a substantial proportion of these conditions are categorized as work-related musculoskeletal disorders (WMSD), which arise or are exacerbated by exposures inherent to specific job tasks and working conditions. In industrial settings, where physical workloads are often intense and repetitive, risk exposures such as awkward postures, sustained static loading and forceful or repetitive upper-limb activity markedly increase the likelihood of developing WMSD, particularly affecting the neck, shoulders and lower back (3, 5).

Manufacturing workers represent a particularly vulnerable group, as their tasks commonly involve repetitive manual operations, constrained postures, limited variation in movement and insufficient opportunities for recovery. Epidemiological evidence highlights Despite decades of research demonstrating the benefits of conservative WMSD management (especially through physical activity, patient education and self-management strategies) evidence-based recommendations are not consistently implemented in routine occupational practice (6–8). Barriers such as limited time, resource constraints, and variability in organizational support often restrict the delivery of structured preventive programs in workplaces.

International clinical guidelines underscore the importance of exercise, education and behavioral change strategies as core components in managing musculoskeletal complaints, with documented benefits in reducing pain, enhancing functional capacity and improving long-term outcomes (3, 8). Multimodal interventions combining exercise and education have shown clinically meaningful improvements in pain, mobility, psychological well-being and functional performance across a variety of musculoskeletal conditions, including knee osteoarthritis, shoulder instability and other chronic MSD (1, 9–11) Nevertheless, research focused specifically on occupational cohorts (especially those employed in manufacturing and textile industries) remains limited.

Technological innovations and the growing expansion of digital health offer new opportunities to address this gap. Hybrid rehabilitation models that integrate in-person instruction with digital health support tools, such as mobile applications, remote monitoring systems and adaptive exercise platforms, have demonstrated feasibility, acceptability and potential benefits in diverse clinical and educational contexts (9, 11, 12). These digital health platforms facilitate personalized exercise progression, improve adherence, and extend therapeutic engagement beyond face-to-face sessions. Although promising results have been reported in community and clinical populations, their implementation in industrial workplaces, where organizational, ergonomic and productivity-related constraints differ substantially, remains is still underexplored.

Textile workers face ergonomic demands associated with repetitive upper-extremity tasks, prolonged standing and high-paced production workflows. Recent epidemiological studies focusing specifically on the textile industry report a high prevalence of work-related musculoskeletal disorders, with rates frequently exceeding 60–70% among workers exposed to repetitive tasks and prolonged static postures (13, 14). The most commonly affected regions include the neck, shoulders and lower back, largely due to repetitive upper-limb movements, sustained forward-flexed postures and limited task variability (15). In addition, psychosocial factors such as high production demands, low job control and time pressure have been identified as relevant contributors to symptom persistence and chronicity in textile workers (16). Despite this, research on tailored interventions specifically designed for this sector remains scarce. Few studies have examined workplace-based programs that integrate health education with structured exercise strategies or explored the potential of hybrid delivery models that support accessibility and adherence in demanding industrial environments. The limited availability of rigorous evidence on preventive or health-promoting approaches for textile industry workers highlights the need for targeted, context-specific interventions grounded in educational frameworks that encourage safe movement, active lifestyles and sustained physical engagement.

Against this background, workplace-embedded multimodal programs that deliver both onsite health education and supervised exercise, together with home-based exercise supported by a digital app, may represent a feasible and scalable strategy to address MSD burden in manufacturing environments. Delivering the educational components and supervised exercise sessions directly at the worksite enhances accessibility and ecological validity, while the app-guided home-exercise program facilitates continuity and adherence. This integrated onsite and home model aligns with contemporary models of occupational health promotion and has the potential to mitigate symptoms, improve functional capacity and strengthen workers’ self-management skills.

The aim of this study is to examine the changes associated with an eight-week multimodal program (combining onsite health education workshops and supervised exercise with a home-based app-supported exercise intervention) on musculoskeletal pain, physical function, psychosocial outcomes, work ability and productivity among textile workers with WMSD, using a pre–post quasi-experimental design.

By integrating workplace-based education with supervised and app-guided exercise, this study seeks to provide a feasible and scalable approach to reduce the burden of musculoskeletal disorders in industrial settings. Based on current evidence on multimodal interventions and digital health support, we expect that participation in the program will be associated with reductions in musculoskeletal pain and improvements in physical function, psychosocial outcomes, work ability and productivity at post-intervention and follow-up assessments.

## 2 Methods

### 2.1 Study design

This study will use a quasi-experimental, single-arm, pre–post design to evaluate outcomes over time following a multimodal exercise and health education intervention among manufacturing workers. The study protocol is reported in accordance with the SPIRIT guidelines and was registered at clinicaltrials.gov (NCT07360626).

All eligible participants will receive an eight-week multimodal intervention comprising a combined exercise and health education program delivered using a hybrid format (face-to-face and online sessions). Participants will undergo a comprehensive baseline assessment performed by the research team to enable individualized exercise prescription. The same assessment protocol will be repeated immediately after intervention and at a one-month follow-up to evaluate within-participant changes in study outcomes.

### 2.2 Study setting

This study will be conducted at the facilities of a medium-sized textile manufacturing company located in Zaragoza, Spain. The company operates within the industrial textile sector and includes several production-related departments such as manufacturing, quality control, logistics, and administrative services.

The intervention will be implemented within the workplace environment, facilitating integration of the program into routine occupational activities. Face-to-face assessments and workshops will be carried out on-site during working hours, while remote exercise sessions will be delivered through a mobile application.

### 2.3 Participants

Participants will be manufacturing and office workers employed at the participating textile company, of both sexes, aged between 18 and 65 years, who voluntarily agree to participate and meet the eligibility criteria.

Inclusion criteria:

- Current employment contract with the participating company throughout the intervention period and the one-month follow-up.
- Age between 18 and 65 years.
- Availability to attend the scheduled sessions.

Exclusion criteria:

- Severe or specific musculoskeletal conditions contraindicating exercise.
- Current pregnancy.
- Concurrent physiotherapy treatment.
- Temporary work disability due to illness during the intervention period.
- Insufficient physical capacity to perform workshops or guided exercises.
- Cognitive or communication impairments.

### 2.4 Recruitment

Recruitment will take place at the facilities of the participating textile manufacturing company. Study information will be prepared by the research team and disseminated internally by the company’s management.

To prevent any perceived pressure from participating, company management will not be involved in participant identification, eligibility screening, informed consent, data collection or data analysis. Participation will be entirely voluntary, and workers will be explicitly informed that their decision to participate or decline would have no impact on their employment conditions or relationship with supervisors. In addition, the workers’ council (employee representative committee) was informed about the study and agreed with the study procedures, ensuring employee representation and oversight throughout the research process.

Informational meetings will be held across all work shifts to explain the study objectives and procedures and to address participants’ questions.

Following the informational meetings, workers will receive written information about the study, including contact details for the principal investigator. Those expressing interest will contact the investigator, be screened for eligibility, and provide written informed consent prior to inclusion.

**Figure 1:**
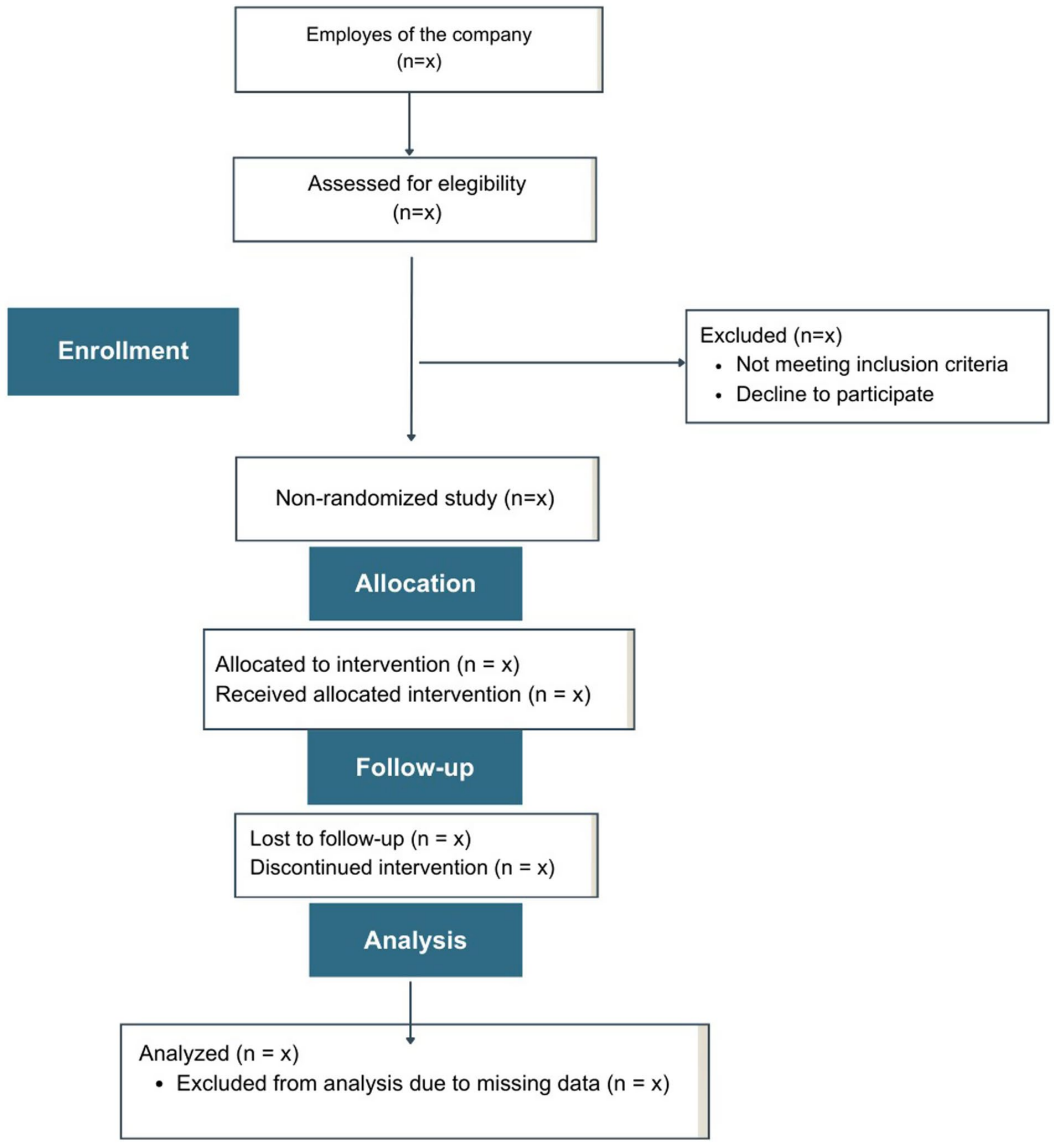
Flowchart of the study design.

A TREND-compliant flow diagram of participant enrolment is shown in Figure 1.

### 2.5 Interventions

The intervention will be delivered over an eight-week period by members of the research team, including qualified physiotherapists and physical activity and sports science professionals. It will combine exercise and health education in a hybrid format (face-to-face and app-based), consisting of eight on-site educational workshops and a structured app-based exercise program.

#### 2.5.1 Face-to-face workshops

Participants will attend eight structured group workshops, delivered one per week, during working hours at the company’s facilities. To optimize accessibility and participation, sessions will be scheduled separately for each work shift (morning, afternoon, and night).

An initial introductory workshop will be delivered before starting the exercise program to ensure safe participation. It will include instruction on the use of exercise materials, guidance on adjusting exercise intensity, and basic guidance on muscle activation and correct execution of basic exercises.

Workshops will follow structured educational programs using standardized materials to ensure consistency across groups. The content will address:

- Pain mechanisms and coping strategies, with emphasis on self-efficacy.
- Principles of strength and aerobic exercise.
- Mobility training.
- Stress management and relaxation techniques.
- Active rest strategies during the working shift.
- Sleep hygiene and healthy lifestyle habits.
- Review sessions aimed to clarify doubts and reinforce learning through group discussion.

During these sessions, participants will also receive guidance on the digital exercise component and may ask questions about prescribed exercises.

#### 2.5.2 App-based exercise program

Participants will complete two weekly exercise sessions via the RehBody digital platform, a mobile-based software that enables remote prescription, delivery and monitoring of exercise programs through video demonstrations, written instructions and automated session tracking. RehBody is a paid platform; however, for the purposes of this study, access will be provided free of charge to all participants for the duration of the intervention as part of their participation in the research. Participants will receive individual login credentials and instructions on how to access and use the platform during the initial workshop.

Individualized programs will be assigned and updated based on baseline assessments, and investigators will monitor adherence and progression through the platform’s control panel. Participants will be able to access the program via smartphone or computer. To support engagement, the platform will also include visual progress tracking and structured exercise logging. Furthermore, participants will be able to contact the research team through the platform to receive feedback if they have doubts about exercise execution or experience discomfort.

The program will be designed to promote general mobility, scapular and spinal stability, and upper- and lower-limb strength, with a particular focus on preventing injuries and strengthening the joints most affected by the company’s work tasks, including the shoulders, wrists, elbows, and hips. Each session will follow a structured progression, beginning with a warm-up of mobility exercises and core activation, and continuing with a main phase targeting key muscle groups. Exercise intensity and complexity will progressively increase, from simple bilateral movements to complex unilateral and multi-joint tasks. The volume and complexity of exercises will be adjusted according to participants’ perceived difficulty and tolerance, and any repetitions that cause excessive discomfort will be modified to maintain therapeutic benefit while minimizing symptom exacerbation. The structure and progression of the exercise program are detailed in Table 1.

**Table 1:** Structure and progression of the 8-week exercise program.

Phase	Weeks	Main objective	Target region	Exercise characteristics	Volume and intensity	Progression criteria (App-based)
Phase 1: adaptation	1–2	Restore motor control and neuromuscular adaptation	Deep trunk stabilizers, scapular stabilizers, hip stabilizers	Low-load, bilateral, predominantly single-joint exercises; low coordination demand	2–3 sets; 10–12 reps RPE 3–4	≥80% session completion; pain ≤3/10; RPE ≤ 4; No symptom worsening within 24 h
Phase 2: load introduction	3–4	Develop basic strength and joint stability	Shoulder complex; wrist stabilizers; lumbopelvic region	Bilateral multi-joint exercises; increased ROM and core engagement	2–3 sets; 12–15 reps; RPE 4–5	≥80% adherence; Average RPE ≤ 5; No adverse events
Phase 3: dynamic stability and unilateral control	5–6	Enhance dynamic stability and unilateral neuromuscular control	Rotator cuff, scapulothoracic musculature, hip abductors and extensors	Unilateral multi-joint exercises; increased balance and coordination demands	3 sets; 8–12 reps; RPE 5–6	≥80% Adherence; RPE 5–6 without excessive fatigue; Positive tolerance feedback
Phase 4: functional integration and movement efficiency	7–8	Integrate strength into functional movement patterns	Upper limb–trunk–hip kinetic chain	Functional, task-oriented multi-joint exercises; higher coordination, control, and movement efficiency demands	3 sets; 8–10 reps; RPE 6–7	≥80% adherence; RPE ≤ 7; Self-reported readiness to progress

All participants will receive an exercise kit designed to facilitate the completion of home-based sessions, including elastic bands of different resistance levels, an exercise mat, a foam roller, and massage balls.

Adherence to the intervention will be monitored using attendance records for face-to-face workshops and usage data from the RehBody application. Participants will be considered adherent if they attend at least 80% of the planned sessions. This adherence threshold will be used to define the per-protocol sample for the primary analysis.

TIDieR alignment: The intervention description follows the TIDieR checklist, specifying what, who, how, where, when, how much, and individualization/progression.

### 2.6 Outcomes

#### 2.6.1 Sociodemographic data

Sociodemographic data and work-related characteristics will be collected before the intervention using an *ad hoc* questionnaire, including age, sex, job seniority, and work shift. This data will be used to describe and support the interpretation of the study findings.

#### 2.6.2 Primary outcome

*Pain intensity:* Pain intensity will be measured using the Numeric Rating Scale (NRS), an 11-point validated self-reported measure widely used in musculoskeletal and occupational health research. Participants will be asked to rate their average musculoskeletal pain on a scale ranging from 0 (no pain) to 10 (worst imaginable pain) during face-to-face assessment sessions.

The NRS has demonstrated good reliability, validity, and responsiveness to change in musculoskeletal populations. Scores are commonly interpreted as: mild pain (1–3), moderate pain (4–6), and severe pain (7–10) (17).

The MCD for the NRS in musculoskeletal pain has been estimated at 1.5–2 points, which will be used to interpret the clinical relevance of pre–post changes (18). The primary outcome will be the change in NRS scores from baseline to post-intervention.

### 2.7 Secondary outcomes

*Musculoskeletal symptoms:* Musculoskeletal symptoms will be assessed using the Standardized Nordic Musculoskeletal Questionnaire (NMQ) developed by Kuorinka et al. (19) This questionnaire assesses the presence of pain or discomfort in nine body regions (neck shoulder, elbows, wrists/hands, upper-back, hips/thighs, knees and ankles/feet). Participants will indicate whether they have experienced musculoskeletal symptoms in each body region during the preceding 12 months and the preceding 7 days, and whether these symptoms have resulted in limitations in normal work activities.

The NMQ will allow the assessment of the prevalence, anatomical distribution, and work-related functional impact of musculoskeletal symptoms in this occupational population, as well as their changes following the intervention. The Spanish version of the NMQ has demonstrated adequate reliability and content validity, supporting its use in Spanish-speaking working populations (20, 21).

*Muscle strength:* Upper-limb muscle strength will be assessed using a handgrip dynamometer (Takei Grip-D dynamometer) to measure maximal handgrip force (kg), following standardized testing procedures. Participants will perform the test in a seated position with the elbow flexed at 90°, and the highest value of three trials will be recorded.

Handgrip strength is a reliable and valid indicator of general muscle strength and functional health, and it has been extensively used in occupational and clinical populations (22, 23). Reference values adjusted for age and sex will be used for interpretation. The test demonstrates excellent test–retest reliability and concurrent validity, with high intraclass correlation coefficients reported across populations.

*Functional Capacity:* Functional capacity will be evaluated using the 30-s Sit-to-Stand test which quantifies the number of complete sit-to-stand repetitions performed within 30 s. This measure serves as an indicator of lower-limb strength and functional ability associated with everyday and work-related tasks. The 30s STS test is a commonly used, valid and reliable measure of lower extremity strength and functional performance in populations with chronic musculoskeletal pain and working-age adults.

*Physical activity level:* Physical activity level will be assessed using the short form of the International Physical Activity Questionnaire (IPAQ-SF). This self-administered questionnaire records the frequency and duration of walking, moderate-intensity, and vigorous physical activity performed during the previous seven days (24).

This outcome will be expressed in MET minutes per week and categorized as low, moderate, or high according to established scoring guidelines. The IPAQ-SF has demonstrated acceptable reliability and validity for population-based studies and has been validated in Spanish-speaking populations (25, 26).

*Work ability:* Work ability will be evaluated using the Work Ability Index (WAI), a multidimensional self-administered questionnaire that evaluates perceived work ability in relation to job demands, health status, and mental resources. The total score ranges from 7 to 49 points, with higher scores reflecting better perceived work ability, and is classified into four categories: poor (7–27), moderate (28–36), good (37–43), and excellent (44–49) (27). The WAI has been widely used in occupational health research and has demonstrated good psychometric properties, including validation in Spanish working populations.

#### 2.7.1 Psychosocial and health-related outcomes

*Sleep quality:* Sleep quality will be evaluated using the Pittsburgh Sleep Quality Index (PSQI), a validated self-administered instrument that assesses sleep patterns and disturbances over the previous month across seven components subjective quality, latency, duration, efficiency, disturbances, use of sleep medication, and daytime dysfunction). The PSQI generates a global score ranging from 0 to 21 points, with higher scores indicating poorer sleep quality; a score above 5 is commonly used to identify clinically relevant sleep disturbance (28). The Spanish version of the PSQI has demonstrated adequate reliability and validity in adult populations (29).

*Depression, anxiety and stress:* Symptoms of anxiety, depression, and stress will be measured using the Depression, Anxiety and Stress Scale (DASS-21), providing separate subscale scores (0–42) for each domain. Higher score indicates greater symptoms of severity. Interpretation thresholds for each subscale are normal, mild, moderate, severe, and extremely severe. The MCD is approximately 5–6 points per subscale (30). The DASS-21 has shown good psychometric properties and has been validated in Spanish populations (31).

*Health-related quality of life:* Health-related quality of life will be measured using the EQ-5D-5L a generic questionnaire assessing five dimensions: mobility, self-care, usual activities pain/discomfort, and anxiety/depression, each with five severity levels. It also includes a visual analog scale (VAS) ranging from 0 (“worst imaginable health”) to 100 (“best imaginable health”). Higher score indicates better health status (32). The Spanish version is validated for adult populations (33).

*Work productivity and absenteeism:* Work productivity, activity impairment, and absenteeism will be assessed using the Work Productivity and Activity Impairment questionnaire (WPAI). The instrument provides four outcomes: absenteeism (% of work time missed), presenteeism (% impairment while working), overall work productivity loss, and activity impairment in daily activities outside work. Higher percentages indicate greater impairment (34). The WPAI has been validated in Spanish populations (35).

In addition to clinical and work-related outcomes, selected process indicators will be descriptively reported to contextualize feasibility aspects of the intervention in this occupational setting. These will include recruitment rate, attendance to face-to-face workshops, adherence to the app-based exercise sessions, and retention at follow-up. These indicators will be intended to inform interpretation of the results and guide future-controlled studies.

All outcome variables and their corresponding assessment time points are detailed in Table 2.

**Table 2:** Outcome measures.

Outcome measure	Baseline (T0)	Post-intervention 8 weeks (T1)	Follow-up 12 weeks (T2)
Primary outcome			
Pain intensity (NRS)	✓	✓	✓
Secondary outcomes			
Musculoskeletal symptoms (NMQ)	✓	✓	✓
Upper-limb muscle strength (Handgrip dynamometry)	✓	✓	✓
Functional capacity (30-s Sit-to-Stand test)	✓	✓	✓
Physical activity level (IPAQ-SF)	✓	✓	✓
Work ability (WAI)	✓	✓	✓
Sleep quality (PSQI)	✓	✓	✓
Depression, anxiety and stress (DASS-21)	✓	✓	✓
Health-related quality of life (EQ-5D-5L)	✓	✓	✓
Work productivity and absenteeism (WPAI)	✓	✓	✓
Sociodemographic and work-related variables	✓	—	—
Process/feasibility indicators			
Recruitment rate	—	✓	—
Workshop attendance	—	✓	—
Adherence to app-based exercise sessions	—	✓	✓
Retention at follow-up	—	—	✓

### 2.8 Data collection procedure

Baseline assessments will be conducted face-to-face at the company’s facilities. Questionnaire-based outcomes will be completed on paper under the supervision of a member of the research team, while physical tests will be administered by qualified professionals following standardized protocols.

All outcome measures, except for baseline sociodemographic data, will be reassessed immediately after completion of the intervention and at a one-month follow-up, using identical assessment protocols, time schedules and, where possible, by the same assessor.

All collected data will be anonymized and coded using a unique identification number assigned to each participant. Personal identifying information will be stored separately from research data and will be accessible only to the principal investigator. Paper-based questionnaires will be kept in locked cabinets at Universidad San Jorge, and electronic data will be entered into a password-protected database hosted on secure institutional servers compliant with European data protection regulations (GDPR and Spanish Organic Law 3/2018). Data entry will be double-checked to minimize transcription errors, and range and consistency checks will be performed prior to statistical analysis. Data will be retained for 5 years following study completion and then securely destroyed.

**Figure 2:**
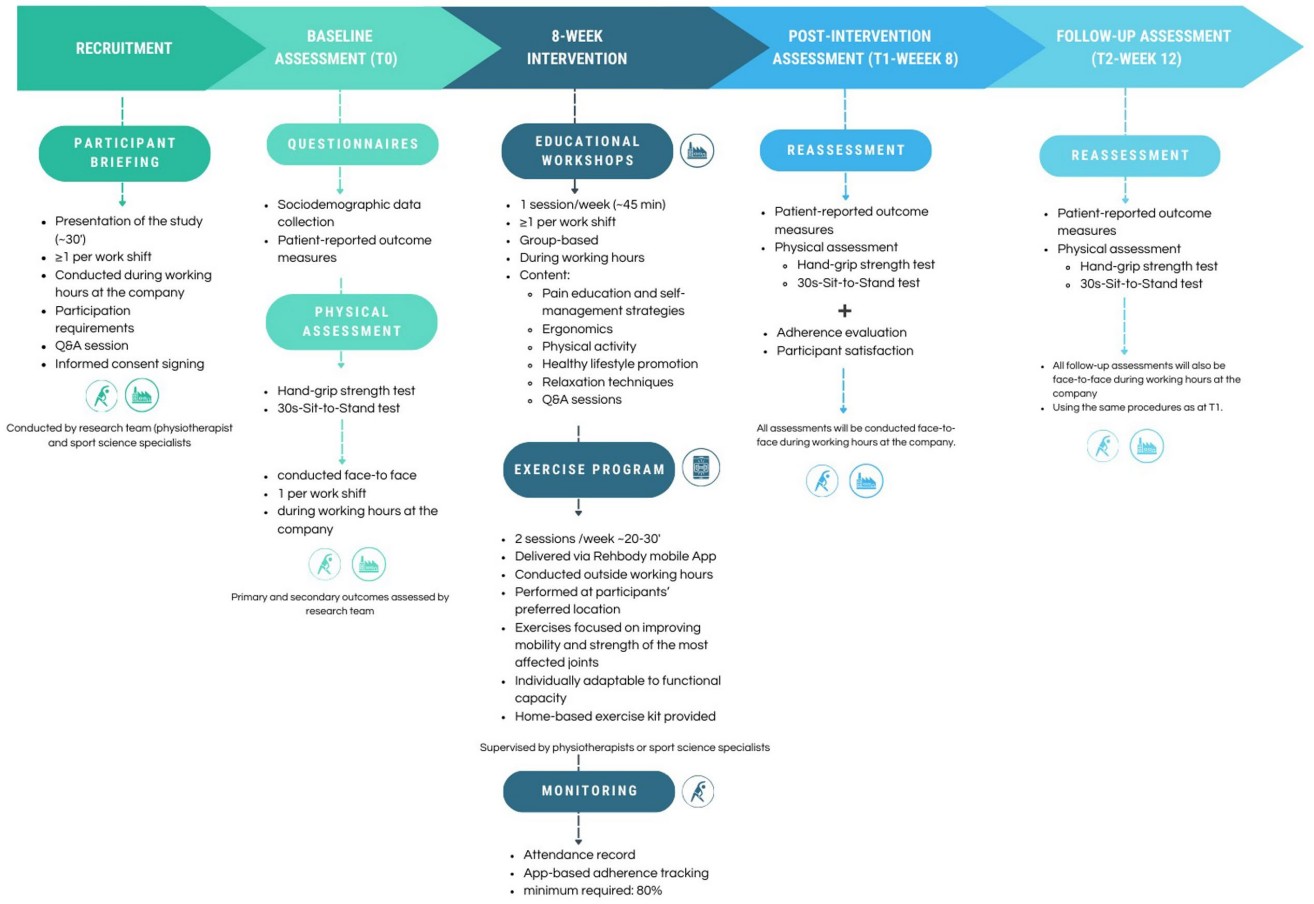
Study timeline.

Figure 2 presents the study timeline and key procedural details.

### 2.9 Sample size

Based on previous studies conducted in industrial worker populations evaluating similar interventions, a minimum clinically important difference of 1.5 points on the NRS for pain was assumed, together with a standard deviation of 2.5 points (36, 37). Using a two-sided *α* level of 0.05 and a statistical power of 80%, the required sample size for a paired-sample comparison was estimated to be 27 participants (Cohen’s d = 0.60). Accounting for an anticipated 20% attrition rate, the final adjusted target sample size was set at 33 participants. The computation was performed using Jamovi (version 2.7.6; www.jamovi.org), specifically the power analysis module for paired-sample *t*-tests.

### 2.10 Statystical analysis

The statistical analysis of the quantitative data will be performed using IBM-SPSS Statistics version 29 (IBM Corp., Armonk, NY, United States). Data will be expressed as the mean and the standard deviation with a 95% confidence interval or as the median and the interquartile range. The Shapiro–Wilk test will be used to assess normality.

Given the longitudinal design with three assessment points (baseline, post-intervention, and one-month follow-up), changes over time will be analysed using repeated-measures analysis. When parametric assumptions are met, a repeated-measures ANOVA will be conducted, with post-hoc pairwise comparisons adjusted using the Bonferroni correction.

For non-parametric data, the Friedman test will be used to assess overall changes over time, with post-hoc pairwise comparisons conducted using Wilcoxon signed-rank tests, and *p*-values adjusted using the Bonferroni correction.

Effect sizes and 95% confidence intervals will be reported to quantify the magnitude of observed changes, using eta squared (η^2^) for ANOVA analyses and Kendall’s W for Friedman tests, in addition to *p*-values.

The primary analysis will follow a per-protocol approach, including only participants who attended at least 80% of the planned intervention sessions, as defined by the adherence criteria.

Statistical significance will be set at *p* < 0.05.

### 2.11 Ethics and dissemination

The study protocol was approved by the Universidad San Jorge Ethics Committee (N° 7/1/25–26) and the Research Ethics Committee of Aragón (PI25/463).

The findings of the study will be disseminated through academic conferences and peer-reviewed journals. No public access to the dataset is planned due to confidentiality considerations. No individual-level data will be shared with the company. Only anonymized and aggregated results will be reported.

## 3 Discussion

This study protocol proposes a workplace-embedded, multimodal intervention that combines health education and a digitally supported exercise program to promote musculoskeletal health among textile manufacturing workers. The proposed approach is innovative in this context, as it integrates structured face-to-face educational workshops with an app-based exercise intervention delivered within a real industrial environment. By adopting a hybrid delivery model, the intervention aligns with contemporary principles of occupational health promotion and adult learning, emphasizing experiential, participatory and self-management-oriented strategies (38, 39). Delivering the program directly at the workplace is expected to enhance accessibility, ecological validity, and engagement, particularly in settings where time constraints and workload often limit participation in conventional preventive programs (40).

The selection of textile manufacturing workers is particularly relevant due to the specific ergonomic demands of this sector, which include highly repetitive upper-limb tasks, sustained static postures, manual material handling, limited task variability, and production-related constraints that are strongly associated with work-related musculoskeletal disorders. In this context, textile manufacturing represents a particularly appropriate setting for workplace-embedded and hybrid intervention strategies, where feasibility, adherence, and integration into production-driven environments are critical considerations. Accordingly, the present study addresses an underserved occupational population using a hybrid, workplace-embedded intervention specifically designed to enhance feasibility, adherence, and scalability.

WMSD are among the leading global causes of disability and work limitations, with substantial socioeconomic impact across working populations (41). Repetitive tasks, awkward postures, and sustained static loads are strongly associated with high prevalence of neck, shoulder, and low back disorders in manufacturing and assembly-line work (16, 42). Workers with MSD are also associated with significant negative impacts on productivity, presenteeism, absenteeism, and quality of life, contributing to substantial economic and organizational costs (43). Evidence shows that workers with musculoskeletal pain are more likely to experience reduced work performance and functional limitations, even when absenteeism is not markedly increased (44, 45).

Prior workplace evidence demonstrates that structured, on-site strengthening programs are associated with reductions in neck and shoulder pain among industrial workers, supporting the feasibility and added value of delivering key intervention components directly within the work environment (46). Such findings are consistent with broader research (47) showing that exercise and education constitute first-line strategies for the prevention and management of WMSD, with workplace-based physical exercise programs linked to reductions in pain, analgesic use, and work absence, as well as reductions in productivity loss and improvements in physical capacity (48).

However, despite these benefits, such interventions are not consistently implemented in routine occupational practice. This implementation gap has been attributed to organizational and contextual barriers, including limited management support, lack of time during work shifts, insufficient resources, low worker adherence, and the prioritization of short-term productivity over preventive health measures (41, 49, 50).

In response to these implementation challenges, hybrid intervention formats that combine limited face-to-face input with predominantly online content have emerged as a promising strategy to improve feasibility, adherence, and scalability in occupational settings. Digital and remotely delivered workplace health interventions allow greater flexibility in scheduling, reduce time and organizational constraints, and facilitate integration into daily work routines, thereby addressing some of the main barriers to traditional on-site exercise programs (51). Moreover, hybrid models enable the delivery of structured exercise, education, and behavioral components at lower cost and greater reach, which may increase the likelihood of long-term adoption in manufacturing and industrial environments (49, 51, 52). Recent umbrella and systematic reviews of workplace interventions indicate that multicomponent and self-monitoring supported strategies can meaningfully increase physical activity/engagement at work, while digital delivery enhances reach key considerations for industrial settings with shift work and production targets, key considerations for textile manufacturing environments characterized by shift work and production pressures (53).

Nevertheless, many workplace programs continue to focus primarily on isolated ergonomic adjustments or brief educational sessions, with limited integration of structured exercise and behavioral components, which may limit their long-term impact (54). Reviews of workplace interventions highlight the need for more comprehensive, multimodal approaches that combine ergonomics, exercise, and behavioral strategies to address musculoskeletal disorders among manufacturing workers, underscoring the relevance of workplace-embedded and hybrid intervention models such as the one proposed in the present study (50).

In conclusion, our study protocol builds on prior evidence that supervised, workplace-based strengthening reduces industrial neck/shoulder pain, embedded programs improve physical capacity and pain outcomes when adherence is achieved, and app-guided exercise may enhance adherence in real-world occupational settings. This protocol specifically addresses the need for tailored preventive strategies in textile manufacturing, providing a coherent rationale for a hybrid, workplace-embedded model.

### 3.1 Strengths and limitations of this study

This study describes a workplace-embedded, multimodal intervention combining health education and a digitally supported exercise program, specifically designed for textile manufacturing workers, which enhances ecological validity and real-world applicability.

The absence of a control group was a pragmatic decision driven by contextual and organizational constraints of the workplace setting. The intervention was implemented at the company level during working hours, and randomization of workers or staggered (stepped-wedge) implementation was not feasible due to production requirements, shift organization, and the company’s preference to offer the program equally to all interested employees. Therefore, a single-arm pre–post design was considered the most appropriate approach for this preliminary evaluation.

The hybrid delivery model (face-to-face workshops plus app-based exercise) may improve accessibility, adherence and scalability of musculoskeletal health promotion programs in industrial settings with time and workload constraints.

The use of validated outcome measures across physical, psychosocial and work-related domains allows a comprehensive evaluation of intervention effects beyond pain alone.

The quasi-experimental pre–post design without a control group limits causal inference and does not account for potential temporal or external confounding factors.

The relatively small sample size and single-center recruitment may limit the generalizability of the findings to other occupational sectors or industrial contexts.

## 4 Conclusion

This study protocol describes a structured, workplace-embedded multimodal intervention combining health education and app-supported exercise tailored to the specific demands of textile manufacturing workers. By integrating face-to-face workshops with a digitally supported home-based program, the proposed approach aims to enhance accessibility, adherence and sustainability in a real-world occupational setting.

The study is expected to contribute to preliminary hybrid workplace interventions for the prevention and management of work-related musculoskeletal disorders in industrial populations. If supported by findings from future controlled studies, this model may offer a scalable and transferable strategy for improving musculoskeletal health, functional capacity and work-related outcomes, supporting the implementation of comprehensive occupational health promotion programs in similar industrial environments.
